# Spontaneous Rupture of Hepatocellular Carcinoma With Fatal Outcome in a Patient Taking Direct-Acting Antivirals

**DOI:** 10.7759/cureus.46638

**Published:** 2023-10-07

**Authors:** Youness Dendane, Ghizlane Kharrasse, Abdelkrim Zazour, Hajar Koulali, Zahi Ismaili

**Affiliations:** 1 Hepato-Gastroenterology, Digestive Disease Research Laboratory, Mohammed VI University Hospital Center/Mohammed First University, Oujda, MAR; 2 Gastroenterology and Hepatology, Mohammed VI University Hospital Center/Mohammed First University, Oujda, MAR

**Keywords:** liver imaging, spontaneous rupture, hepatocellular carcinoma (hcc), direct anti-viral agents, hepatitis c (hcv)

## Abstract

Hepatocellular carcinoma (HCC) is one of the most common malignant tumors globally. Many complications are attributed to it, including spontaneous rupture, which is a serious and rare complication that can be life-threatening. Managing and detecting this condition might pose challenges, especially when there is no prior history of liver cirrhosis or tumor. We report on a 57-year-old man followed as an outpatient for chronic hepatitis C who presented to the emergency department for abdominal pain with abdominal distention and jaundice, occurring two months after treatment by direct-acting antiviral (DAA). He was not known to have a liver tumor on the ultrasound performed before the start of treatment. Therefore, the diagnosis of tumor rupture was not very clear. The evolution was fatal, and death occurred quickly. Although the association between DAA treatment and hepatocarcinogenesis and its possible complications is unknown, close monitoring by high-performance imaging is probably required in patients under DAA.

## Introduction

Roughly 700,000 people succumb to hepatocellular carcinoma (HCC) annually across the globe, ranking it as the third leading cause of cancer-related deaths [1]. The majority of HCC cases (85%-95%) arise in the context of cirrhotic liver [2]. Spontaneous rupture is a rare complication, and its incidence seems to be declining through screening and advancements in early detection [3,4]. The optimal treatment approach for ruptured HCC continues to be a subject of ongoing discussion and debate. The therapeutic choices should be based on factors like underlying liver function, tumor stage, and feasibility of resection. Therefore, managing ruptured HCC requires a multidisciplinary approach. The impact of direct-acting antiviral (DAA) on precancerous conditions or existing cancer nodules remains unclear. This case report will lead us to explore the available data and potential hypotheses shedding light on the perplexing association between DAA treatment and HCC occurrence and rupture.

## Case presentation

A 57-year-old man was admitted to the emergency department of our hospital for acute abdominal pain with abdominal distension and jaundice. He was followed as an outpatient for chronic viral hepatitis C diagnosed two months ago and treated with DAA. The only radiological examination carried out before the start of treatment was an abdominal ultrasound, which did not reveal a tumor or signs of chronic liver disease or portal hypertension. At admission, physical examination revealed a conscious patient, hemodynamically and respiratory stable, icteric, with a distended abdomen, without signs of portal hypertension or hepatocellular insufficiency. Streaks of bright red blood were found at digital examination.

Laboratory tests revealed the following findings: hemoglobin at 11.9 g/dL then 9.9 g/dL after 24 hours, platelets at 204,000/µL, predominantly direct hyperbilirubinemia at 153 mg/L associated with cytolysis and hepatic cholestasis: serum alkaline phosphatase 468 U/L, gamma-glutamyl transferase 473 U/L, serum aspartate aminotransferase 385 U/L, serum alanine aminotransferase 102 U/L; an albumin level of 30 g/L, prothrombin time (PT) at 49%, factor V at 81.40% (7,000-12,000), alpha-fetoprotein (AFP) > 20,000.00 ng/mL, inflammatory markers were elevated with white blood cells at 13,550/µL and C-reactive protein (CRP) at 80 mg/L, renal function was preserved and the blood ionogram was without abnormality. The ascites puncture showed macroscopically hematic fluid.

**Figure 1:**
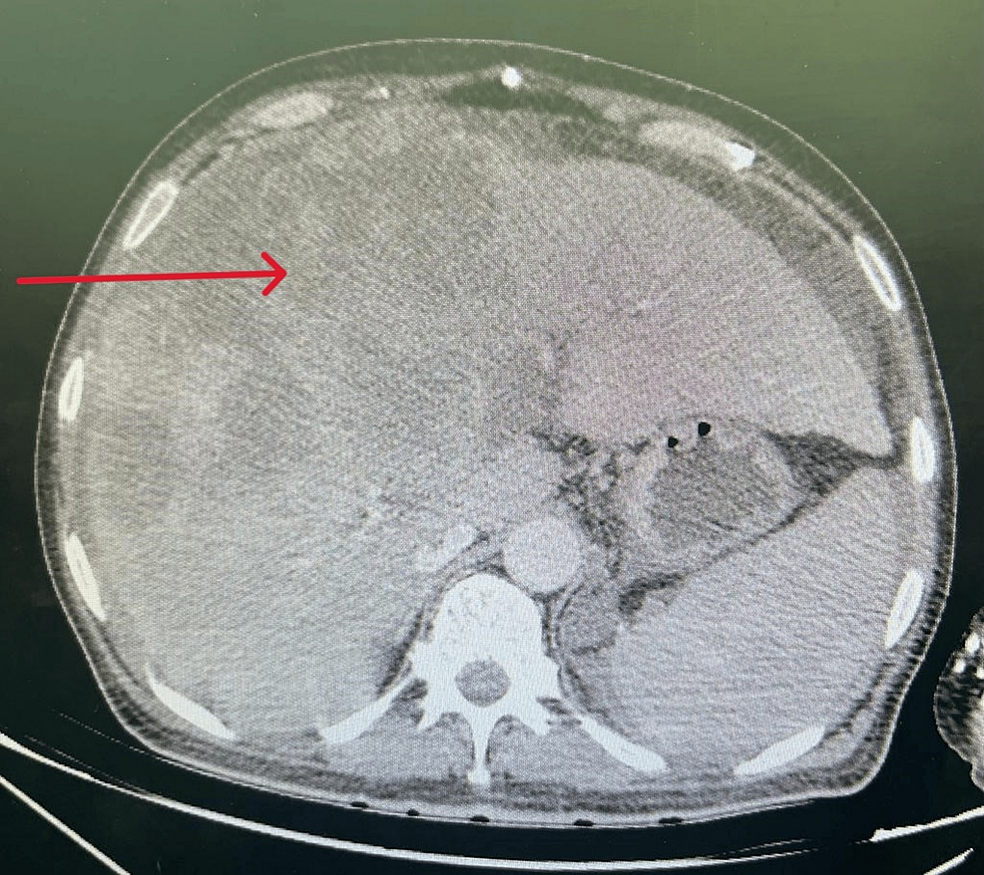
Liver tumor mass occupying segments IV and VIII, with exophytic development.

**Figure 2:**
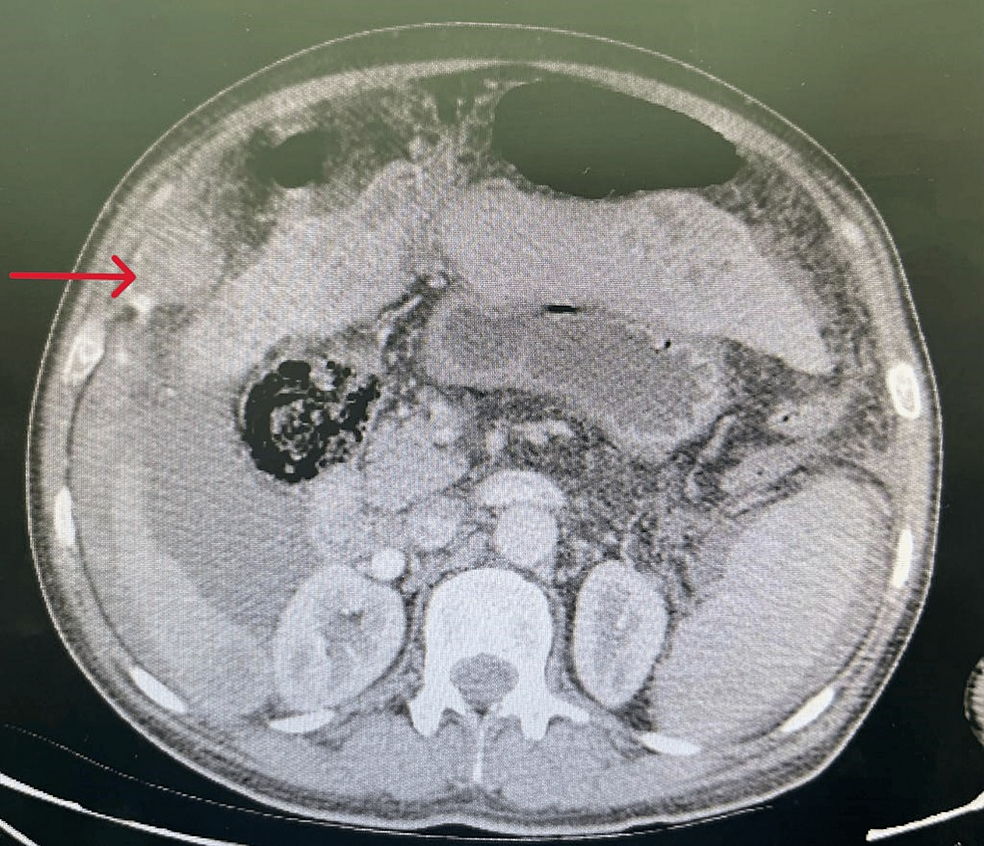
Extravasation of the contrast product in the arterial phase.

**Figure 3:**
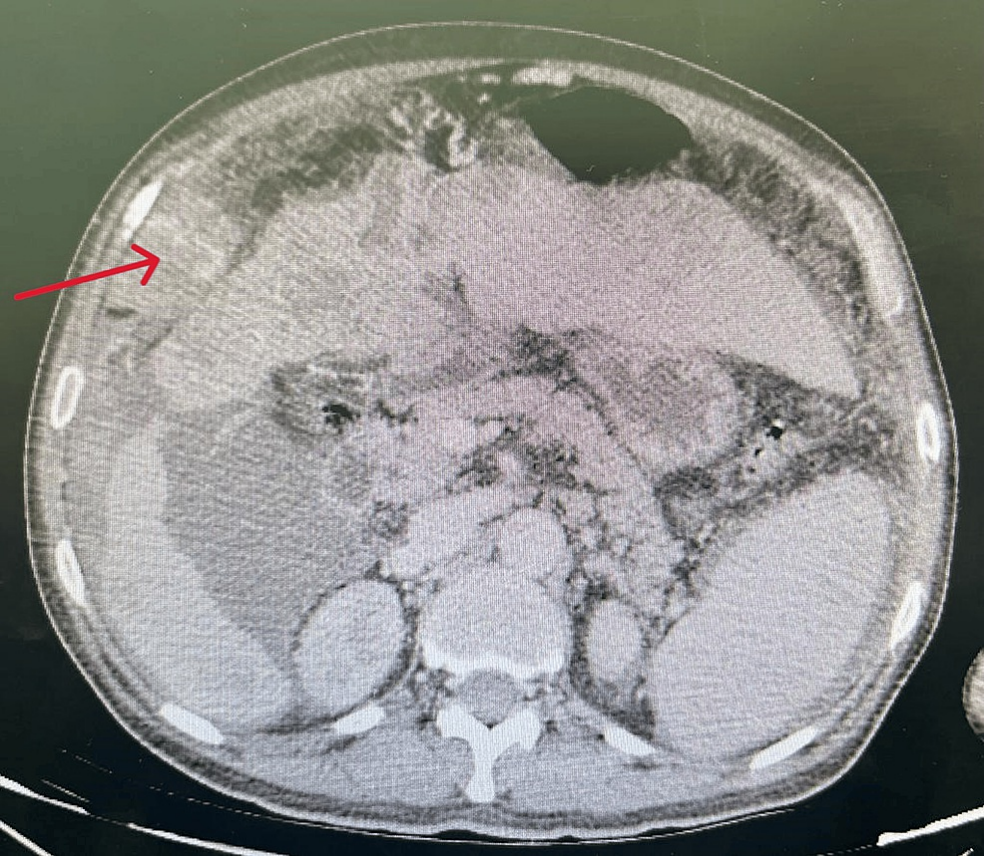
Increased extravasation of the contrast product in the venous phase.

An ultrasound was performed which was non-contributory, motivating the realization of a CT scan that showed extravasation of the contrast product in the arterial phase, increased in the venous and late phase related to active bleeding from an arterial branch that feeds the hepatic tumor mass occupying segments IV and VIII measuring 138 x 140 mm, with exophytic development, presenting a washout, first suggesting HCC, metastatic to the lymph nodes, lungs, and peritoneum, hematoma of the right flank associated with a large hemoperitoneum, chronic liver disease with thrombosis of the portal trunk extended to its right and left branches (Figures 1-3).

**Figure 4:**
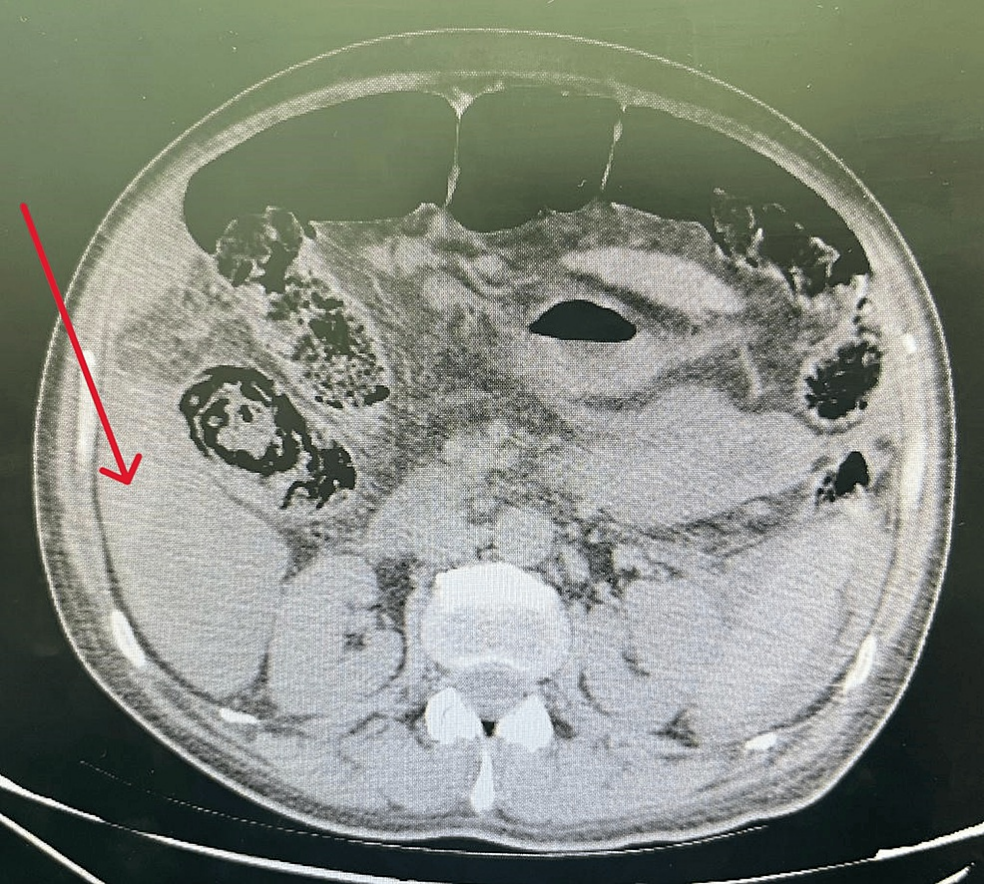
Intraperitoneal effusion of hematic density peri- and subhepatic.

The evolution was marked by the rapid onset of hypotension with impaired consciousness, followed by cardio-respiratory arrest that was not recovered after resuscitation measures.

## Discussion

The risk of HCC in HCV-infected patients is increased by 15- to 20-fold, with the annual incidence of HCC being estimated at 1% to 4% in cirrhotics over a 30-year period [5]. Based on the estimates from 2020, liver cancer, largely dominated by HCC, is the sixth most commonly diagnosed cancer and the third most common cause of cancer death [6]. Spontaneous rupture is the third most common cause of death related to HCC, following tumor progression and liver failure [7]. It occurs in 3%-15% and is associated with worse short- and long-term prognosis [8-10].

The mechanism behind the spontaneous rupture of HCC remains incompletely understood. Nevertheless, researchers have identified several factors that may contribute to the rupture and hemorrhage of HCC. One crucial factor is the characteristics of the tumor itself. Ruptured HCC is more frequently observed in nodular and massive forms, while it is rare in diffuse HCC. The size, location, and growth rate of the tumor significantly influence the likelihood of spontaneous rupture. Tumors with a diameter exceeding 5-7 cm, protruding more than 1 cm from the liver's surface, and located in specific segments of the liver (II, III, IV B, VI) are independent risk factors for rupture and hemorrhage. Rapid tumor growth, necrosis, and local invasion can elevate intratumoral pressure and venous congestion, potentially leading to vessel atresia, vascular dysfunction, and increased fragility of the vessels [4,11,12]. Liver cirrhosis is another key factor associated with HCC hemorrhage. After the rupture and hemorrhage of HCC, the liver's condition worsens due to hypoperfusion injury, resulting in reduced synthesis of coagulation factors and coagulation dysfunction [13,14].

Regarding the clinical presentation, acute abdominal pain is the most common symptom, occurring in 66%-100% of cases [15-17]. Shock at presentation can be observed in 33%-90% of patients [10,15-19], and abdominal distension has been reported in 33% of cases [20]. Liver failure can occur in the acute phase, affecting 12%-42% of patients [4]. Abdominal paracentesis, which documents hemoperitoneum, is a reliable test for diagnosing ruptured HCC in up to 86% of clinically suspected cases [15]. However, with advancements in active surveillance of HCC in cirrhosis and improved imaging modalities, the rate of diagnosing HCC rupture has shown gradual improvement. On ultrasound, approximately 66% of cases of HCC rupture present as a hyperechoic area surrounding the tumor [21]. Moreover, it can be confirmed by computed tomography (CT) scan with high sensitivity and specificity, according to previous studies [22-24], making it the modality of choice in the diagnosis of HCC hemorrhage. Nevertheless, a considerable portion (20%-33%) of cases were diagnosed only during emergency exploratory laparotomies in previous studies [10,22].

The optimal treatment approach in the management of HCC rupture remains a subject of debate, necessitating a thorough pre-treatment evaluation considering factors like hemodynamic status, liver function, tumor characteristics, and HCC stage to determine the most suitable treatment option. Conservative treatment involves volume resuscitation, blood transfusion, and cardiovascular monitoring. However, the outcomes are generally poor, with high hospital mortality rates and short median survival [25]. Hence, conservative management is limited to moribund patients with poor liver function and advanced tumor stages where other interventions are not feasible. Trans-arterial embolization (TAE) is a less invasive method with a high success rate in stopping bleeding from ruptured HCC during the acute phase [4,22,26-28]. A serum bilirubin level of less than 3 mg/dL is considered a reliable indicator of successful TAE outcomes [29,30]. Surgical hemostasis depends on the patient's hepatic reserve and the extent of liver cirrhosis. Emergency liver resection, though associated with higher mortality and lower success rates compared to staged liver resection, offers the advantage of achieving hemostasis and potentially curative tumor removal [4,10,31]. Other rare techniques of hemostasis include radiofrequency ablation (RFA) and microwave coagulation, both of which have shown potential effectiveness, but more research is needed to understand their long-term outcomes and limitations [32,33]. Liver transplantation is not considered a suitable option for managing ruptured HCC due to the risk of disease recurrence, and it is categorized as an absolute contraindication in the United Kingdom listing criteria for LT in HCC [34].

Otherwise, it is important to give a brief reminder on the treatment of viral hepatitis c, before determining whether or not the new molecules currently recommended are involved in the appearance of HCC and its complications. For over two decades, the mainstay of chronic hepatitis treatment was Interferon (IFN). Although it was utilized widely, its severe adverse effects and limited curative potential raised concerns. Recently, a new class of medications, known as DAA agents, has revolutionized the landscape of hepatitis C treatment. These agents generate minimal side effects and have demonstrated success in curing elderly individuals and patients with cirrhosis. Notably, unlike IFN, DAAs target the virus directly, bypassing the need for host immunity involvement, with consequently an impressively high sustained virological response (SVR) rate.

It was initially believed that DAA treatment would serve as a preventive measure against HCV-related HCC. However, recent reports have taken the medical community by surprise, revealing an unexpected surge in HCC occurrence or recurrence following DAA therapy [35,36]. This conflicting evidence has ignited a controversial debate on whether DAA treatment truly prevents or inadvertently promotes hepatocarcinogenesis. Conversely, there have been two distinct reports that present a different perspective, indicating that DAA treatment might not increase the risk of HCC recurrence [37,38]. These studies propose that the observed HCC cases during or shortly after DAA treatment could be attributed to the natural progression of precancerous lesions or early HCC that might have existed before DAA initiation.

Certainly, and as has already been described in the literature, the size of our patient's tumor, its rapid growth, its location, its exophytic development, and the presence of extensive portal thrombosis are probably all elements that precipitated the occurrence of this fatal complication.

However, the patient's clinical course raises another significant point for discussion: the potential impact of DAAs on precancerous conditions or pre-existing cancer nodules, and their potential association with complications, especially tumor progression and rupture. Although The relationship between DAAs and carcinogenesis is not fully understood. Villanl et al. found that VEGF levels increased during DAA treatment and remained elevated up to the end of treatment, potentially affecting the balance between inflammatory and anti-inflammatory processes and altering antitumor surveillance in the host [39,40]. These high VEGF levels might probably lead to carcinogenesis with tumor hypervascularization. Another mechanism reported by Serti et al. suggests that DAA-mediated clearance of HCV leads to a loss of intrahepatic immune activation by IFN-α, which could allow tumor growth as IFN-α plays a role in inhibiting tumor cell proliferation [41]. Also, Spaan et al. demonstrated that treatment with DAAs resulted in a reduction of viral load in chronic HCV patients, which, in turn, led to decreased levels of NK cell-stimulating cytokines and modifications in the phenotype of NK cells [42]. All these mechanisms might have played a role in the development and rupture of HCC in our patients. The escape from anti-tumor control, at the origin of a local aggressiveness with the invasion of the vascular structures, very rich secondarily to the high rate of angiogenic factors, in particular VEGF, could in large part explain the tumor rupture of our patient.

Although our case might be rare, since no other similar case has been found published in the literature, careful and close monitoring of patients on DAA using cross-sectional imaging is essential, given the risk of carcinogenesis or even fatal complications as is the case of our patient, which can sometimes be missed on ultrasounds.

## Conclusions

The impact of DAAs on precancerous conditions or pre-existing cancer nodules remains uncertain, given the lack of solid argument and the few data published in the literature. Our clinical case associated with our literature review aims to provide valuable insights to guide medical practitioners in making informed decisions about DAA treatment strategies for patients with hepatitis C, ensuring optimal outcomes while minimizing any potential risks related to HCC and its complications. Through careful monitoring of patients using high-performance imaging, future treatment approaches for hepatitis C can be refined, leading to improved safety and effectiveness.
